# Alexithymic traits in adolescents with Anorexia Nervosa

**DOI:** 10.1192/j.eurpsy.2022.973

**Published:** 2022-09-01

**Authors:** I. Nicolau, A. Iotu, M. Leti, L. Andrei, M. Stancu, F. Rad

**Affiliations:** 1 “Prof.Dr. Al. Obregia” Psychiatry Clinical Hospital, Child And Adolescent Psychiatry Department, Bucharest, Romania; 2 Carol Davila University of Medicine and Pharmacy, Child And Adolescent Psychiatry Department, Bucharest, Romania

**Keywords:** Anorexia nervosa, alexithymia, eating disorder, adolescence

## Abstract

**Introduction:**

Alexithymia is a construct which has been described in persons under the autistic spectrum. Besides Autistic Spectrum Disorders, alexithymia nowadays is highly correlated with several psychiatric disorders, among them being Eating Disorders. Several studies suggested a “cognitive-affective” division in the inner experience of patients with Anorexia Nervosa, because of their difficulty in describing, identifying and recognising their own emotions as well as others

**Objectives:**

This study aims to identify how many adolescents diagnosed with Anorexia Nervosa meet the characteristics of alexithymic personality traits and in which domain of these traits they had the most struggles with.

**Methods:**

The study lot includes 34 adolescents diagnosed with Anorexia Nervosa evaluated by a self report survey: Online Alexithymia Questionnaire-G2 (OAQ-G2). The cut-off scores are: 113 and above - correlated with alexithymia, 95-112 - correlated with possible alexithymia and under 94 - insignificant clinical score. We analyzed the result of every subcategory of the questionnaire in order to determine whether there is an area affected more than others.

**Results:**

The sample included 34 patients, female to male ratio 31:3, evaluated in the Department of Child and Adolescent Psychiatry, “Prof. Dr. Alexandru Obregia” Psychiatry Hospital. 29,41% had clinically semnificative scores for alexithymic traits, while 52,94% scored for possibile alexithymia according to the OAQ-G2.

**Conclusions:**

In 82,35% of patients from the lot we identified alexithymic personality traits. The F1 subcategory (difficulty identifying feelings) and F5a (problematic interpersonal process) were the ones that distinguished the alexithymic group from the possible alexithymic one.

**Disclosure:**

No significant relationships.

